# Robust and efficient coding with grid cells

**DOI:** 10.1371/journal.pcbi.1005922

**Published:** 2018-01-08

**Authors:** Lajos Vágó, Balázs B. Ujfalussy

**Affiliations:** NAP-B PATTERN Group, MTA Wigner Research Center for Physics, Budapest, Hungary; Stiftung caesar, GERMANY

## Abstract

The neuronal code arising from the coordinated activity of grid cells in the rodent entorhinal cortex can uniquely represent space across a large range of distances, but the precise conditions for optimal coding capacity are known only for environments with finite size. Here we consider a coding scheme that is suitable for unbounded environments, and present a novel, number theoretic approach to derive the grid parameters that maximise the coding range in the presence of noise. We derive an analytic upper bound on the coding range and provide examples for grid scales that achieve this bound and hence are optimal for encoding in unbounded environments. We show that in the absence of neuronal noise, the capacity of the system is extremely sensitive to the choice of the grid periods. However, when the accuracy of the representation is limited by neuronal noise, the capacity quickly becomes more robust against the choice of grid scales as the number of modules increases. Importantly, we found that the capacity of the system is near optimal even for random scale choices already for a realistic number of grid modules. Our study demonstrates that robust and efficient coding can be achieved without parameter tuning in the case of grid cell representation and provides a solid theoretical explanation for the large diversity of the grid scales observed in experimental studies. Moreover, we suggest that having multiple grid modules in the entorhinal cortex is not only required for the exponentially large coding capacity, but is also a prerequisite for the robustness of the system.

## Author summary

Navigation in natural, open environments poses serious challenges to animals as the distances to be represented may span several orders of magnitudes and are potentially unbounded. The recently discovered grid cells in the rodent brain are though to play a crucial role in generating unique representations for a large number of spatial locations. However, it is unknown how to choose the parameters of the grid cells to achieve maximal capacity, i.e., to uniquely encode the utmost locations in an open environment. In our manuscript, we demonstrate the surprising robustness of the grid cell coding system: The population code realised by grid cells is close to optimal for unique space representation irrespective of the choices of grid parameters. Thus, our study reveals a remarkable robustness of the grid cell coding scheme and provides a solid theoretical explanation for the large diversity of the grid scales observed in experimental studies.

## Introduction

Optimising neuronal systems for efficient processing and representation of information is a key principle for both understanding and designing neuronal circuits [1], but deciding whether a particular neuronal phenomenon reflects an optimisation process is often difficult. Grid cells in the medial entorhinal cortex have been suggested to efficiently represent spatial location of the animal by their spatially periodic firing fields near optimally [2, 3, 4, 5]. However, it remained controversial whether the efficiency of the grid cell code is the result of the precise tuning of the grid parameters [6, 7, 8] or the performance of the system is relatively insensitive to the actual parameter settings [4, 5, 9].

**Fig 1:**
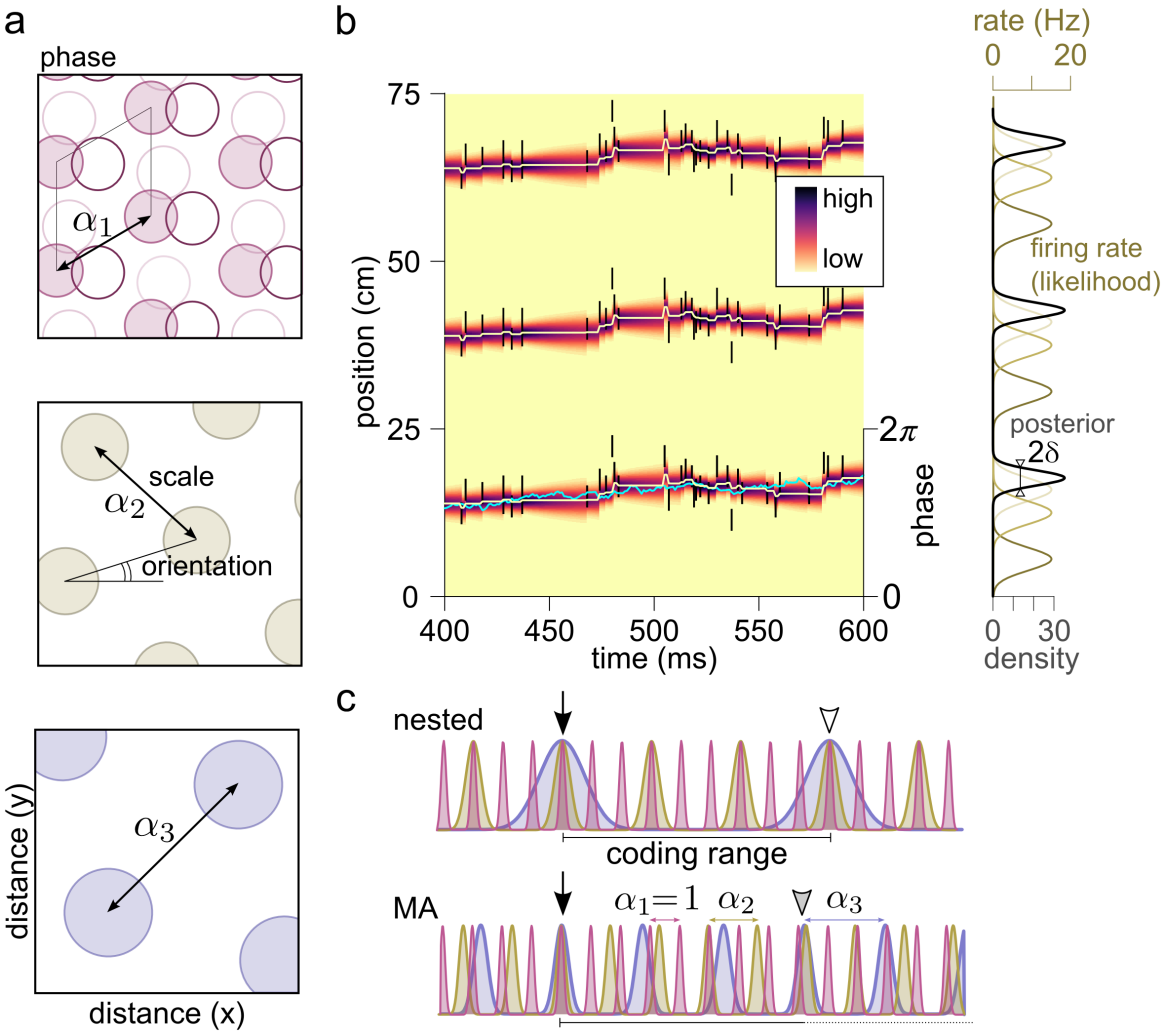
Coding with grid cells. **(a)** Schematic firing fields (circles) of two-dimensional grid cells as function of spatial position. Grid cells are organised into modules: Cells from the same module share the orientation and scale parameter but differ in their spatial phase (top, shades of purple). Different modules have different scale and orientation (top to bottom). **(b)** Grid cell spikes encodes the phase of a module. Spiking of grid cells (black ticks, each spike is shown three times, at the maxima of the cells’ firing rate) from a single module represents the movement of the animal (light-blue line) in a 1 dimensional environment. Since the firing rate of the cells (right, olive) is periodic, the position (left: colormap, right: black) which is represented by the phase of the module is also periodic. The uncertainty of the representation fluctuates over time around a typical value, *δ* (right). **(c)** Grid cell coding schemes. The location of the animal (origin, filled arrow) is jointly encoded by the phases of the different modules in both nested (top) and modulo arithmetic (bottom) codes. Grey and empty arrowheads indicate locations with large or catastrophic interference between the modules, respectively.

Grid cells are spatially tuned neurons with multiple firing fields organised along the vertices of a triangular grid (Fig 1a; [10, 11]). Grid cells of any particular animal are organised into functional modules [12, 13] cells within a module share the same grid scale and orientation, but differ in the location of their firing fields, i.e., their preferred firing phase within the grid period (Fig 1a). Modules form the functional units of the grid representation: The joint activity of all (possibly hundreds of) cells within each module is captured by the (two dimensional) phase of the given module (Fig 1b; [14, 15]) and the relationship between different cells from the same module remains stable across different environments [16], during sleep [17, 18] or after environmental distortions [13]. A given spatial location is represented by the phases of the different modules (‘phase vector’). The representations are unique up to a critical distance above which the coding becomes ambiguous: the phase vectors, and hence the firing rates of all grid cells, become (nearly) identical at two separate physical locations (Fig 1c).

Depending on the magnitude of the critical distance compared to the largest grid scale, two complementary coding schemes have been proposed for grid cells (Fig 1c): In nested coding [4, 6, 8] smaller grid modules iteratively refine the position coding of larger modules and the modules span a wide range of scales. The capacity of nested codes, defined as the ratio of the coding range and the resolution, is exponential in the number of modules. Maximal capacity can be achieved by setting the coding range equal to the maximal grid period and then optimising the resolution by a geometric progression of the grid scales [6]. When the total capacity is utilised to encode locations within the maximal grid scale, catastrophic interference will cause ambiguity in the grid code beyond this distance (Fig 1c).

When the coding is not optimised for a fixed range, the unique combination of the activity of grid modules can encode a potentially unbounded range that can be substantially larger than the scale of the largest module using a modulo arithmetic (MA) code [2, 3, 14] (Fig 1c). In this case the grid periods can be similar in magnitude (e.g., co-prime integers or a geometric progression with a relatively small ratio). However, it is not known under what conditions the MA coding system can achieve exponential capacity [3, 14], and how robust is the capacity against the choice of the grid periods or neuronal noise.

Here we develop a novel approach to study the capacity of the grid coding system that is based on Diophantine approximations, i.e., approximation of real numbers by rational numbers. First, we apply the technique to study coding with two grid modules. We show that the capacity of the system is extremely sensitive to the number theoretic properties of the scale ratio between the modules. Next, we generalise our approach to the case of multiple modules, and show both analytically and numerically that the exponential capacity of the grid cell coding system can be achieved using the MA coding scheme. Finally, we demonstrate that when the coding range is constrained by neuronal noise, the capacity of the system is extremely robust against the choices of the scaling of the modules.

## Results

In the first section of the Results we briefly introduce the terminology and define the concepts used throughout the paper. We include this section for completeness, although several ideas presented in this section have been described before, e.g., in [3, 4].

**Fig 2:**
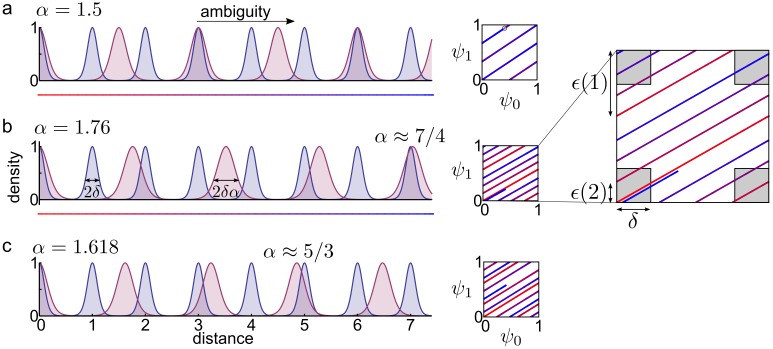
Interference depends on the choice of the scales. **(a)** Interference with rational scale ratio. Left: Representative posteriors (*P*(*x*|**s**)) for two modules with scale 1 and *α* = 3/2. Encoding becomes ambiguous at distance 3 from the origin where perfect interference occurs (3 = 2*α*). Right: Phase plot of the two modules, with the colour (red to blue) encoding the distance from the origin (see the coloured line below the left panel). Perfect interference occurs when the phase-curve overlaps with itself. **(b)** Interference with *α* = 1.76 …, which is close to 7/4 and therefore leads to strong interference at distance 7. Right: Interference occurs when the distance between two neighbouring segments of the phase curve becomes smaller than the limit set by the neuronal noise (grey squares of side *δ* around the origin, see inset). Note, that both grids are around phase 0.3 at the distance 2.3 without interference. **(c)** Interference with *α* = *σ* ≈ 1.618, which is the golden ratio. Interference still becomes stronger at larger distances, (e.g. at distance 5, since $\sigma \approx \frac{5}{3}$). Interference in grid codes is related to the approximation of irrationals with rational numbers having small denominators (see text for further details). Right: Interference is inevitable since the phase space has a limited volume.

We investigate grid cell population codes along a linear trajectory as the one dimensional results extend to two (or higher) dimensions without difficulty, at least for axis aligned grid modules (Methods) [3, 4]. The periodic population activity of module *i* can be summarised by its spatial phase

$$\begin{array}{c} \psi_{i}\left(x\right)≔\left(x\;\text{mod}\;\alpha_{i}\right)/\alpha_{i}\;\in \left[0,1\right), \end{array}$$

which depends on the position (*x*) and the scale of the module (*α*_*i*_, Methods) with *α*_0_ = 1, which means that distances are expressed in the unit of the smallest grid period. We assume, that, without loss of generality, at the spatial origin all modules are in their 0 phase. Spikes of the neurons in module *i*, represent the spatial location of the animal with a maximum error *δ* *α*_*i*_ (i.e., with the same 0.01 ≤ *δ* ≤ 0.2 phase error for all modules; Methods; [8]). Ambiguity occurs if the phase difference *ϵ* between the modules is smaller than *δ* at integer distance *ℓ* from the origin (Figs 1c and 2b, inset, Methods):

$$\begin{array}{c} \epsilon (\ell )=\left|\right|\psi_{1}\left(\ell \right)-\psi_{0}\left(\ell \right)||=\left|\right|\psi_{1}\left(\ell \right)\left|\right|, \end{array}$$ (1)

where ||*ψ*|| means distance from the nearest integer.

**Fig 3:**
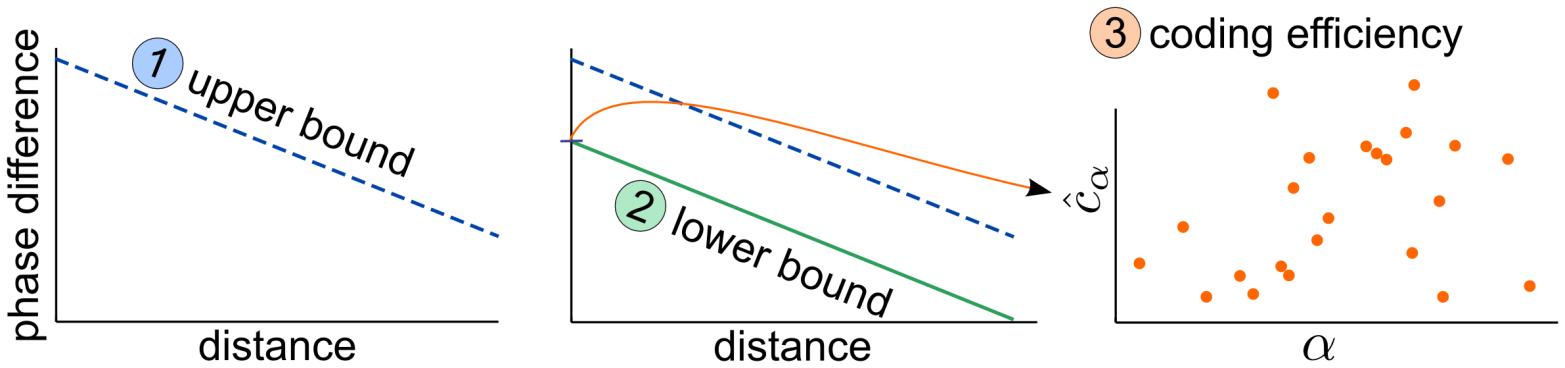
Logical steps of the argument. First, we provide an absolute upper bound on the phase difference in the function of the distance, that is linear in log-log scale (left). Second, we show, that for certain scales a lower bound also exists (middle). Third, we characterise the efficiency of the scales (*α*) by their offset, *c*_*α*_ (right).

To analyse the coding properties of the grid cell system, we follow the same three logical steps both in the two module and in the multi-module case (Fig 3). First, we show the existence of an upper bound on how the maximal phase difference *ϵ*(*ℓ*) between the modules decreases with the distance. Intuitively, this upper bound expresses the fact that interference between the modules necessarily becomes stronger at larger distances. Second, we demonstrate that for appropriately chosen scale ratios a lower bound on the phase difference also exists and is parallel with the upper bound (Fig 3). For these scale choices catastrophic interference is avoided until a critical distance, that depends on the noise level in the system. Importantly, the slope of the bounds depends only on the number of modules, but not on the choice of the scale parameters. Therefore the efficiency of the scale choices (the magnitude of the critical distance) can be characterised by the offset parameter, *c*_*α*_ (defined below), associated with the lower bound. Thus, our third step is to estimate *c*_*α*_ for various choices of the scale parameter *α*.

Our analytic derivations provide an estimate for the asymptotic performance of the system that is valid in the low-noise limit. The main advantage of our approach is that it provides strict bounds on the achievable coding efficiency that can be used as a metric to evaluate the efficiency of different scale choices at realistic noise levels, two or more modules alike. As we found using numerical simulations, these bounds can be approached with random scale choices at realistic levels of noise and number of modules.

### Coding is extremely sensitive to the scale ratio with two modules

We can formalise the problem of interference between two modules as having a pair of integers *k* and *ℓ* with *ℓ* ≈ *kα*, meaning that module 2 (with scale *α*) is close to being in phase 0 at distance *ℓ*, which would cause ambiguity between the coding of the spatial point *ℓ* and the origin. This is formally identical to the number theoretic question of the approximability of the scale *α* ≈ *ℓ*/*k* with rationals having numerator *ℓ*, also known as Diophantine approximations (Fig 2b and 2c).

Hurwitz’s theorem [19, 20] states that for all irrational numbers *α* > 1 there are infinitely many relative primes *k*, *ℓ* such that the error of the approximation, defined as

$$\begin{array}{c} \epsilon (\ell )=|k-\ell /\alpha |, \end{array}$$ (2)

is smaller than the upper bound:

$$\begin{array}{c} \epsilon (\ell )<\frac{1}{\sqrt{5}}\frac{1}{\ell }. \end{array}$$ (3)

Note that the approximation error *ϵ*(*ℓ*) (Eq 2) is the same as the phase difference between the modules, defined in Eq 1, since *ψ*_2_(*ℓ*) = [*ℓ*/*α*] mod 1 = |*k* − *ℓ*/*α*| for an appropriately chosen integer *k* (Fig 2b and 2c). We call *ϵ*(*ℓ*) ‘approximation error’ only when we are talking about approximating irrationals with integer ratios while in the context of grid cells we will call *ϵ*(*ℓ*) the ‘phase difference’.

**Fig 4:**
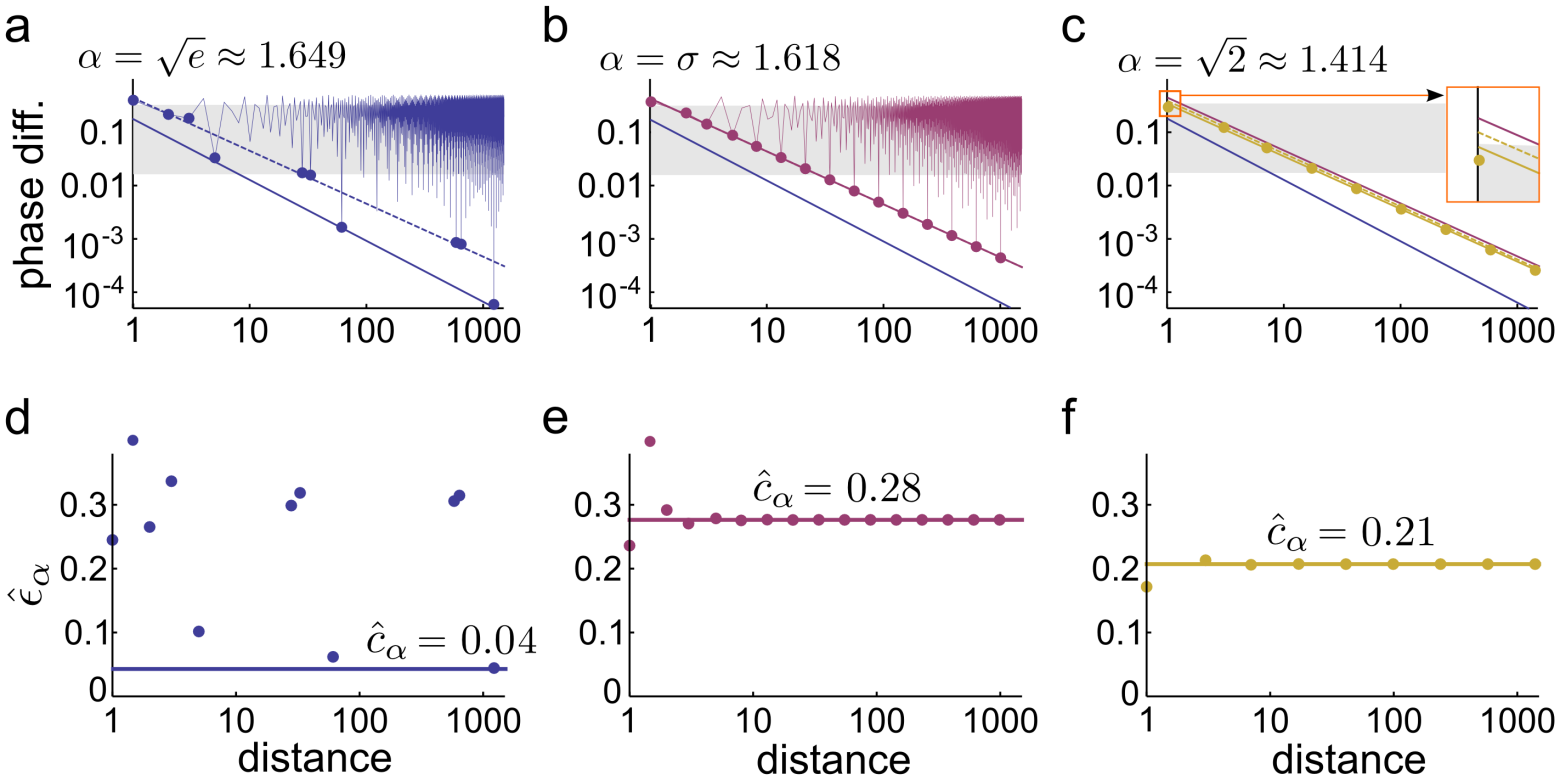
Coding efficiency in two modules. **(a-c)** The phase difference in the function of distance for different values of *α*. The zigzag line indicates the phase difference (PD) at all integer distances, circles indicate record low PD. Dashed line shows the theoretical upper bound of the PD, solid line shows the numerical fit on the lower bound (allowing finitely few exceptions at low *ℓ*). Note, that the lower and the upper bound coincides in b. Also note the 1/*ℓ* scaling of PD for algebraic scale ratios (b-c). Grey shading indicates the range of PD smaller than the neuronal noise, (1 + *α*)*δ*. **(d)-(f)** The scaled phase difference (${\hat{\epsilon }}_{\alpha }\left(\ell \right)$, Eq 7) for different distances and scale ratios. The highest constant under which there are only finitely few values of ${\hat{\epsilon }}_{\alpha }\left(\ell \right)$ at small distances estimates ${\hat{c}}_{\alpha }$. The value of ${\hat{c}}_{\alpha }$ is slightly higher for the golden ratio (e) than for $\sqrt{2}$ (f), and much larger than for non-algebraic numbers (d, $\alpha =\sqrt{e}$, ${\hat{c}}_{\alpha }=0.04$).

Applied to the grid cells, Hurwitz’s theorem provides an upper bound on how the phase difference between the modules shrinks with the distance. Specifically, the theorem states that there are infinitely many integer distances *ℓ*, where the phase difference is smaller than *ϵ*(*ℓ*) ∝ 1/*ℓ* (Fig 4a–4c, dashed lines), implying that on the long run interference can not be avoided no matter how carefully we choose *α*. This is a fundamental upper bound on the efficiency of coding with grid cells.

The critical distance where the phase difference *ϵ*(*ℓ*) leads to interference, and the representation of the position becomes ambiguous, depends on the noisiness of the two modules, *δ* and *αδ*. Interference occurs if there is a spatial point *x* for which both |*x* − *kα*| < *αδ* and |*x* − *ℓ*| < *δ* for integers *k*, *ℓ*, or equivalently, if |*kα* − *ℓ*| < (1 + *α*)*δ*. Hence, by the definition of *ϵ*(*ℓ*) (Eq 2) the coding is ambiguous near *ℓ* if and only if

$$\begin{array}{c} \epsilon (\ell )<\frac{1}{\alpha }\left(1+\alpha \right)\delta . \end{array}$$ (4)

Therefore, no matter how we chose *α*, we can expect ambiguity at distances *ℓ* from the origin if the noise in the system is larger than the upper bound on efficiency provided by Eq 3, i.e.

$$\begin{array}{c} \ell >\frac{\alpha }{\sqrt{5}\left(1+\alpha \right)}\frac{1}{\delta }, \end{array}$$ (5)

that is, at distance of order 1/*δ*. Consequently, it is impossible to code position with two modules better than this bound.

The question arises then whether the above theoretical bound is achievable, at least for some appropriately chosen *α*. The answer is yes, namely the upper bound in Eq 3 is sharp for the golden ratio $\alpha =\sigma ≔\frac{\sqrt{5}+1}{2}\approx 1.618$. Practically, this also introduces a limit on *ϵ*(*ℓ*), saying that the phase difference between the modules remains always larger than a specific lower bound:

$$\begin{array}{c} \epsilon (\ell )>(\frac{1}{\sqrt{5}}-\varepsilon )\frac{1}{\ell } \end{array}$$ (6)

except for a couple of small distances, even for arbitrary small *ε* > 0 [19] (Fig 4b).

It may sound strange that there are finitely many exceptions, but in our simulations we found only a few instances with *ℓ* being small (Fig 4e). Therefore, if the ratio of the two grid modules equals the golden ratio then the phase difference between the two modules is guaranteed to be larger than the lower bound defined by Eq 6. Since *ε* can be arbitrarily small, the lower bound for the golden ratio approaches the theoretical upper bound (Eq 3) and *σ* is an optimal choice for the scale ratio to avoid interference in case of two modules. To give a geometric picture, the golden ratio guarantees approximately uniform coverage of the phase space for both short and arbitrarily long distances (Fig 2c, right).

However, it turns out that there are many good choices [20]: for any algebraic integer *α* of order 2 (i.e. irrational which is a root of a polynomial of degree 2 with integer coefficients, see Methods) there exists a maximal positive constant *c*_*α*_ > 0 such that

$$\epsilon (\ell )\geq c_{\alpha }\frac{1+\alpha }{\alpha }\frac{1}{\ell }$$ (7)

holds except for a couple of small distances (Fig 4c–4f, [21]). Hence, from Eqs 4 and 7 we see that the representation is unambiguous whenever *c*_*α*_(1 + *α*)/(*α* *ℓ*) > *δ*(1 + *α*)/*α*, that is up to

$$\begin{array}{c} \ell \leq L_{\text{max}}≔\frac{c_{\alpha }}{\delta } \end{array}$$ (8)

for all *δ* which is small enough. This last condition on the magnitude of the noise is only needed to exclude the possible exceptionally small *ℓ* distances in Eq 6, which in practice is not a crucial condition (Fig 4d and 4e).

The constant *c*_*α*_ is the single parameter that determines the critical distance up to which encoding is unique (coding range) as well as the information rate of the system (Methods). Therefore, we use *c*_*α*_ to compare the efficiency of different choices of *α* (Fig 4d–4f). We have already noted that for the golden ratio the lower and the upper bounds (Eqs 3 and 7) coincide (Fig 4b), but the critical distance may be larger for some *α* even if the corresponding lower bound on the phase difference is weaker, since the upper bound also depends on *α* (Eq 5).

We estimated the value of *c*_*α*_ for various scale ratios at different noise levels (Methods). Unlike for algebraic numbers, ${\hat{c}}_{\alpha }$ of real numbers depends on the distance range used for the estimation, which we controlled by setting different intervals for *δ* in the simulations.

**Fig 5:**
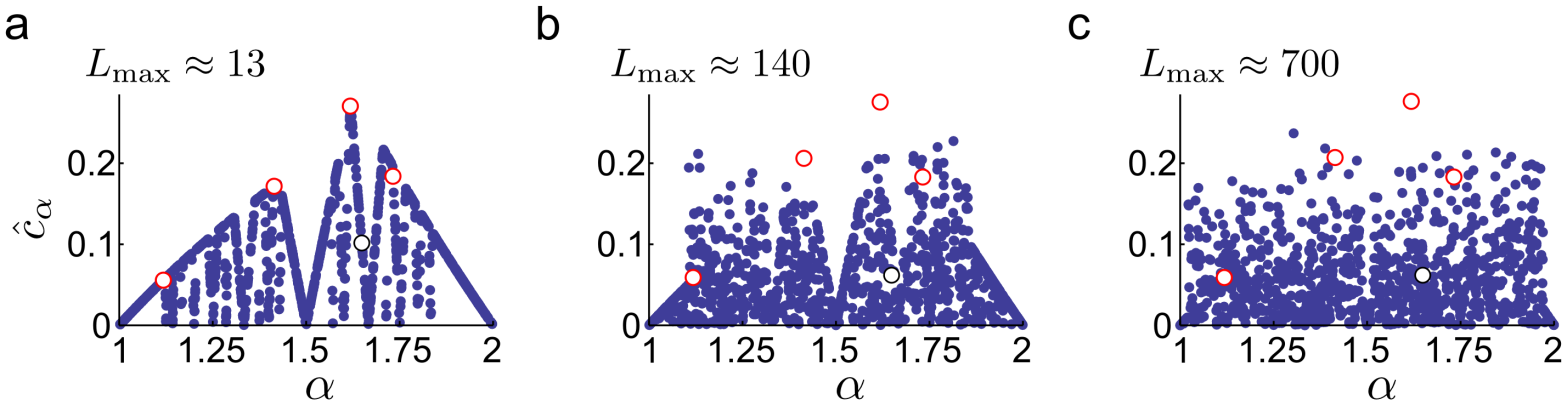
Approximate *c*_*α*_ values as a function of *α*. The values are shown for 1000 *α* randomly selected from the interval (1, 2). The *c*_*α*_ of algebraic ($\sqrt{2}$, $\sqrt{3}$, *σ*, *σ* − 1/2) and non-algebraic ($\sqrt{e}$) irrationals are also shown in red and black, respectively. We estimated *c*_*α*_, based on Eq 8, as *c*_*α*_ ≈ inf{*L*_max_ *δ* ∣ *δ*_min_ < *δ* < *δ*_max_}, for different noise ranges [*δ*_min_, *δ*_max_] in the three panels: (a) 0.05 < *δ* < 0.2, (b) 0.005 < *δ* < 0.05, (c) 0.001 < *δ* < 0.01. Approximate *L*_max_ values are indicated on the top of the panels for *α* = *σ*.

Our simulations confirmed that *σ* is the best scale ratio choice in case of two modules with ${\hat{c}}_{\alpha }=0.28\approx \frac{\sigma }{\sqrt{5}\left(1+\sigma \right)}$, but also showed that, on both short and long run, ${\hat{c}}_{\alpha }$ is extremely sensitive to the choice of *α* (Fig 5): in case of a small error in the tuning of *α*, the efficiency can drop substantially and ${\hat{c}}_{\alpha }$ becomes practically 0, implying that in the immediate neighbourhood of the optimal *α*, there are close to pessimal grid cell configurations. This is because the lower bound on the phase difference (Eq 7) requires *α* to be an algebraic number, and in an arbitrary small neighbourhood of any algebraic number there are (infinitely) many non-algebraic numbers, i.e., transcendental numbers ($\alpha =\sqrt{e}$, Fig 4a) or rational numbers (*α* = 3/2, *c*_*α*_ → 0, Fig 2a). As non-algebraic irrational numbers can be much better approximated with rationals than algebraic numbers, non-algebraic grid scale ratios will lead to much stronger interference between the two modules, but only at distances moderately large compared to the scale of the modules (Fig 5).

The extremely rough landscape of *c*_*α*_ renders optimisation for *α* an especially difficult problem: it is very unlikely that a biological system would be able to find the global optimum for the scale ratio of two grid modules and a relatively small mistuning from a local optimum could significantly deteriorate the efficiency of the system. Therefore, at least in the case of two modules, it seems to be impossible to achieve asymptotically optimal scale ratio for the grid cells.

### Generalisation to multiple modules

**Fig 6:**
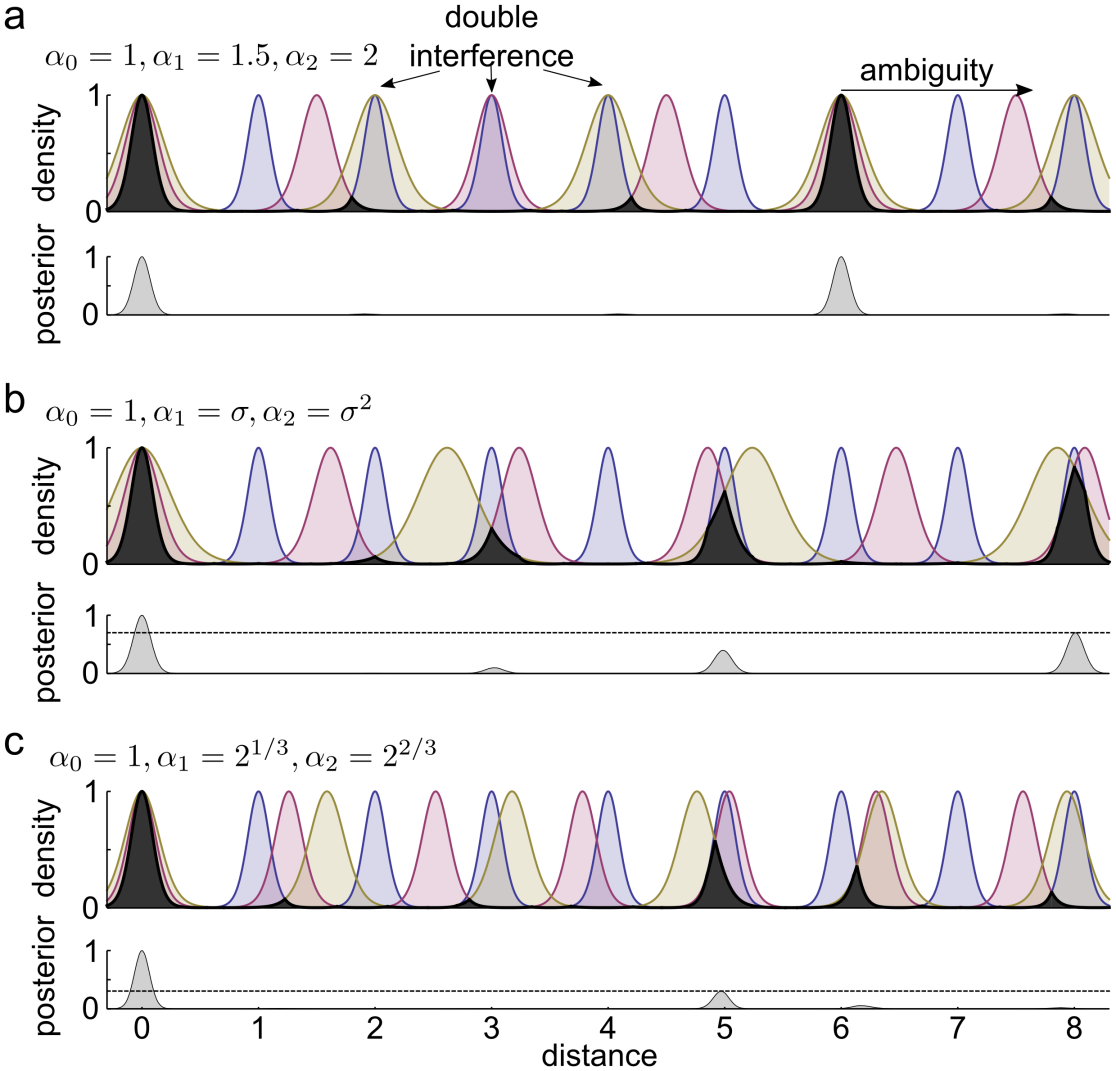
Interference of *M* = 3 modules with different choice of scales. **(a)** Top: Posterior densities for three modules with rational scale ratios. The overlap between the modules is shown in black, its height indicates the interference of the three modules as a function of distance from the origin. The representation becomes ambiguous only if all 3 modules interfere, as at distance 6. Bottom: Ambiguity in position coding quantified by the multi-modality of the combined posterior. **(b)** Posterior densities for three modules with pairwise optimal scale ratios. The scales are 1 (blue), *σ* (red), and *σ*^2^ (olive), where *σ* is the golden ratio. As we have more modules (3) than the order of *σ* (2), wherever any two modules interfere with each other, then they interfere with the third as well: at distance 8 the three peaks almost coincide. **(c)** The same as in (b) for scales 1, 2^1/3^, 2^2/3^, powers of a third order algebraic number. Although pairwise interference can be very strong between any pairs (e.g. at distances 5, 6.2 and 8), the total interference is substantially lower than in panel b (bottom).

To derive the general solution for *M* grid modules, we focus on a set of 1-dimensional grids with scales *α*_0_ = 1 < *α*_1_ < ⋯ < *α*_*M*−1_. Spatial representation is unambiguous up to a distance *L* from the origin if there is at least one module for which the phase is significantly different from 0 (Fig 6). Interestingly, avoiding interference between adjacent modules (giving *α* = *σ*) is not a good solution, since it leads to interference between the distant modules wherever the adjacent modules are in close apposition (Methods). The logic of the general solution for multiple modules is the same as in the case of two modules. Here we only state the main results and the technical details of the analysis can be found in the Methods.

First, we show that a similar upper bound exists for the maximal phase difference between the modules. Compared to the two-module case, the bound is weaker when *M* > 2 as the phase difference scales only with 1/*ℓ*^1/(*M*−1)^ ≫ 1/*ℓ* meaning that it ensures simultaneous interference between all modules only at much larger distances.

Second, we found that the upper bound can be satisfied, up to a constant multiplier, $c_{A}$, for algebraic scale ratios (Methods). Specifically, if the scales of *M* modules form a geometric series with common ratio *α* being an algebraic number of degree *M*, the upper bound is tight, meaning that the phase difference does not shrink faster than 1/*ℓ*^1/(*M*−1)^. Intuitively, this scaling indicates that there is always at least one pair of modules for which the phase difference at the integer distance *ℓ* from the origin is larger than the lower bound.

The critical distance *L*_max_ up to which coding is unambiguous can be expressed as (cf. Eq 8):

$$\begin{array}{c} L_{\text{max}}≔{\left(\frac{c_{A}}{\delta }\right)}^{M-1}, \end{array}$$ (9)

for all *δ* which is small enough, where $c_{A}$ and its estimate ${\hat{c}}_{A}$ are defined analogously as in the two modules case (see Methods for the definition). Intuitively, Eq 9 expresses an exponential scaling of the maximal distance uniquely represented by a population of grid cells with the number of grid modules, *M*. The coding range of a particular set of the grid scales, $A=(\alpha_{1},\ldots ,\alpha_{M-1})$, depends on both the noise in the system and on the basis of the exponential $c_{A}$.

Interestingly, the above described geometric sequence of algebraic numbers are the only known explicit examples of badly approximable vectors (to the best of our knowledge). However, it is known that there are much more such vectors which do not form geometric sequences [22], therefore the scale ratio of a well-tuned MA grid cell system does not have to be constant.

The expression about the exponential scaling (Eq 9) is similar to the capacity estimates of Fiete et al. [3] (see their Eq 6) obtained using a combinatorial upper bound and numerical simulations. Importantly, our analytic derivation also provides insight about why certain grid systems are more efficient than others and give examples for efficient grid scales. Moreover, when $c_{A}=0.5$ our formula for the capacity of the grid code becomes identical to the theoretically maximum capacity found in the case of nested coding [4, 6].

In the next sections we first numerically estimate the value of $c_{A}$ for various choices of the grid scales A and then we show that with sufficiently large number of modules $c_{A}$ is guaranteed to approach its theoretical maximum $c_{A}=0.5$ for randomly chosen grid periods.

### Numerical estimation of the $c_{A}$

**Fig 7:**
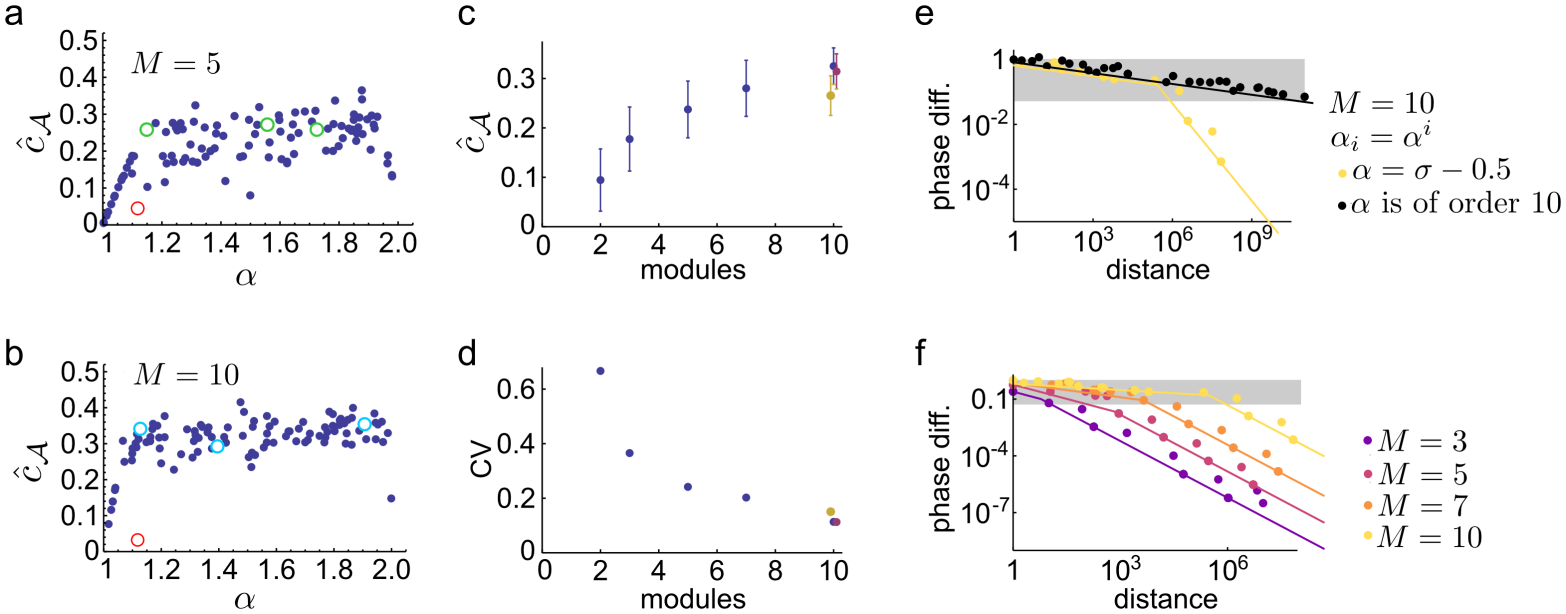
Robustness of the grid code with multiple modules. **(a)-(b)**: Values of ${\hat{c}}_{A}$ estimated for 100 *α* randomly selected from the interval (1, 2) with *M* = 5 (a) and *M* = 10 (b) (see also Fig 5a for *M* = 2). The scales form a geometric series, i.e., $A={\left\{\alpha^{i}\right\}}_{i=1}^{M}$. Red circle indicates ${\hat{c}}_{A}$ for a second order algebraic number *α* = *σ* − 1/2 ≈ 1.118. Green (cyan) shows ${\hat{c}}_{A}$ for 5th (10th) order algebraic numbers, respectively. Noise level is the same as in Fig 5a (0.05 < *δ* < 0.2). **(c)-(d)**: Mean (c) and coefficient of variation (d) of ${\hat{c}}_{A}$ evaluated on the range *α* = {1.1, 1.9}. For *M* = 10 the ${\hat{c}}_{A}$ is also shown for two alternative selection of the scales: if all 10 scales are selected randomly from the interval [1, 2] (olive) and when *α*s form a geometric series perturbed as $A={\left\{\left(1+\varepsilon_{i}\right)\alpha^{i}\right\}}_{i=1}^{10}$, where *ε*_*i*_ are i.i.d. uniform random variables on the range [−0.01, 0.01] (purple). **(e)-(f)** Phase difference (${\hat{\epsilon }}_{A}\left(\ell \right)$, Eq 25) in the function of the distance, *ℓ*. (e) Effect of number theoretic properties with *M* = 10. When *α* is the root of the 10th order polynomial *x*^10^ − *x*^7^ − 1, *α* ≈ 1.12725, *ϵ*(*ℓ*) decays as 1/*ℓ*^1/9^ (black). When *α* is second order, *α* = *σ* − 1/2 ≈ 1.11803, the initial decay is similar, but after a critical distance at *ℓ* ≈ 10^6^ the decay becomes 1/*ℓ* (yellow). **(f)** The critical distance grows with the number of modules (*α*_*i*_ = (*σ* − 1/2)^*i*^). Grey shading in (e-f) indicates the range of phase difference smaller than noise (Eq 26).

We developed an efficient method to numerically estimate the value of $c_{A}$ for various parameter settings that is based on the simultaneous Diophantine approximations of a set of irrational numbers (Methods). Using realistic noise levels we found that, in contrast to the case of two modules, the sensitivity of the coding efficiency to the choice of *α* gradually vanishes when the number of grid modules is increased (Fig 7a and 7b). In particular, with *M* = 10 modules ${\hat{c}}_{A}\in \left[0.2,0.4\right]$ for almost all choices of the grid scales (Fig 7b), both when the scales follow a geometric series with a common scale ratio *α* (Fig 7b) and when all the *M* scales are chosen from the bounded interval (1, 2). We also found that ${\hat{c}}_{A}$ vanishes only for pathological examples such as rational numbers or powers of the second order algebraic number *α* = *σ* − 1/2 ≈ 1.118 (Fig 7a and 7b, red). The only random scale choice that significantly degrades the performance is when *α* ≈ 1 (Fig 7a and 7b) in which case all grid modules have nearly identical spatial scale.

To quantify the sensitivity of the grid system against the choice of the scale parameters we calculated the mean and the coefficient of variation of ${\hat{c}}_{A}$ with random choices of *α* (Fig 7c and 7d). We found, that the average ${\hat{c}}_{A}$ increased monotonically with the number of grid modules indicating that the system’s performance becomes closer to the ideal $c_{A}=0.5$ value as the number of modules increased (Fig 7c). Moreover, the variability of ${\hat{c}}_{A}$ consistently decreased with the number of modules reflecting the improved robustness of the system to the choice of grid periods (Fig 7d). Therefore not only the maximal coding distance increases exponentially with the number of modules, but the basis of the exponential, $c_{A}$, also increases.

To further investigate the mechanisms responsible for the robustness of the system, we numerically evaluated the minimal phase difference between the modules, *ϵ*(*ℓ*), in the function of the distance (Fig 7e and 7f). In line with the predictions of the theory (Eq 25), we found that the phase difference decreased with *ℓ*^−1/(*M*−1)^, i.e., with a small negative power of the distance for *α* being an order *M* algebraic number (Fig 7e, black). For suboptimal *α*-s, the scaling of the phase difference was nearly optimal up to a critical point beyond which the scaling followed the algebraic rank of *α* (i.e., second order *α* scales with 1/ *ℓ*, Fig 7e, yellow). Importantly, this critical point, where the transition occurs between ideal and number theoretical scaling is located at increasingly larger distances when the number of modules is increased (Fig 7f). Therefore, the asymptotic, number theoretical properties of the grid periods have a gradually lower impact on the performance of the system in the distance range limited by the intrinsic variability on neuronal spiking (Fig 7e and 7f, background shading).

These observations suggest that even random scale choices might achieve optimal performance as the number of modules grow. In the next section we make this statement mathematically precise and demonstrate that indeed, ${\hat{c}}_{A}$ approaches its theoretical maximum, 0.5, when the number of modules grow and the scales are chosen uniformly at random from a bounded interval.

### Capacity of non-geometric grid scales

Our number theoretic argument (Eq 9) alone does not imply exponential capacity, since it does not exclude the possibility that the base of the exponential, $c_{A}$, converges to 0 as *M* increases (although we observed the opposite trend, see Fig 7c). In this section we investigate the asymptotic properties of the grid code when the number of modules increases and the relative uncertainty *δ* of the modules remains fixed. Here we only state these results informally, and leave the precise statements and the slightly technical mathematical proof to the Methods.

The main idea behind the proof is that the phase of a given module at particular distance *x* from the origin depends only on the scale of that module, *α*. If the scale is randomly chosen from a bounded interval [1, *α*_max_], then the phase is also a random variable with probability distribution approaching the uniform distribution as the distance increases. Then, the probability of simultaneous interference between *M* modules, that is, the probability of all modules being near phase 0 at some distance *x*, is proportional to the volume of an M-dimensional hypercube, which is *V* = (2*δ*)^*M*^, where the side of the cube is 2*δ*. The ratio of the volume of the hypercube and the unit cube (the number of distinguishable phases) diminishes exponentially with *M*, and the total distance (expressed in units of *α*^0^ = 1) covered without ambiguity is $\frac{2\delta }{V}\propto {\left(\frac{1}{2\delta }\right)}^{M-1}$. Specifically, our statement is, roughly speaking, that if 0 < *δ* < 1/2 is fixed, *M* is large enough, and the module scales are drawn uniformly at random from a not too narrow bounded interval, e.g. from (1, 2), then the representation is unambiguous up to the exponential distance

$$\begin{array}{c} l<{\left(\frac{c_{A}}{\delta }\right)}^{M-1} \end{array}$$ (10)

with probability approaching 1, and $c_{A}$ approaching 1/2. Although the above statement applies only for *M* → ∞, and it does not provide examples for efficient scale choices for finite *M*, we emphasise that this result is stronger than our previous derivation (Eq 9) in four aspects: First, our previous derivation (Eq 9) allowed $c_{A}$ to tend to 0 as *M* increased. Now we showed that this does not happen for random scale choices, rather the value of the constant tends to its theoretical maximum, $c_{A}=0.5$ [6] for large *M* with high probability, confirming our previous numerical results (Fig 7c). Second, one can achieve this nearly optimal performance without increasing the scales exponentially, with the scales chosen from a bounded interval. Third, this almost optimal efficiency is not only reached for some appropriately chosen scales, but for almost all choices. Fourth, near-optimal performance is guaranteed for 2 or higher dimensional grid codes even if the modules are randomly rotated relative to each other or in the absence of long-range coherence within the modules.

Thus, our results demonstrate that no meticulous tuning of the grid scales is required for close to optimal grid system performance.

## Discussion

In this paper we developed a novel analytic technique to investigate the coding properties of grid cells. Using this technique, which is based on Diophantine approximation of real numbers by fractions of integers, we were able to derive several novel and non-trivial properties of the grid cell code. First, we demonstrated that on the long run, the capacity of the system depends heavily and chaotically on the number theoretic properties of the scale ratio between the successive modules. To achieve optimal performance in a system with *M* modules the scale ratio has to be an algebraic number of order *M*. Second, we showed that in the presence of neuronal noise the capacity of the grid code becomes increasingly more robust to the choice of the scale parameters when the number of modules is increased: when *M* > 2, randomly chosen scales perform nearly as well as the optimal scales. Finally, we demonstrated that the capacity of MA and nested grid codes are asymptotically identical (in the large *M* limit), even for randomly chosen scale parameters for the MA codes.

### Exponential coding range

Previous works used specific assumptions to derive exponential coding range for the grid cell coding system: they assumed either a nested coding scheme [5, 6] or presumed that the phase space is covered evenly and that the readout noise in a given module decreases when the number of modules increases [3, 14]. Here we generalised these findings and demonstrated that nested and MA codes have asymptotically equal capacity.

When we studied the capacity of MA codes we realised that achieving uniform coverage of the phase space is not trivial in the case of two modules, but can only be attained with appropriately chosen scales. Specifically, we recognised that approximately uniform coverage of the phase space by the phase curve at arbitrary distances is guaranteed if the scale ratio between the two modules is an algebraic number of order 2. Using our formalism allowed us to generalise this intuition for arbitrary number of grid modules and to demonstrate that even a random choice of grid scales guarantees uniform coverage of the phase space when the number of modules is high.

We also relaxed the assumption of an earlier study [14] that the total amount of the noise remains constant in the grid system even when the number of modules is increased, i.e., the readout error of each module decreases with *M*. Here we derived these results using the more general assumption that the coding precision of each module is independent of *M* and proportional to the scale of the module.

We confirmed our analytical results by extensive numerical simulations regarding the simultaneous interference between grid systems with various choices of the scale parameters. In line with previous results [3, 9], our simulations supported that the grid system is robust to the choice of the scale parameter and that the coding range is exponential in the number of modules.

### Nested coding versus MA code

Although the efficiency of the coding investigated in this paper is slightly worse than that of the optimal nested coding [5, 6], MA codes also have several advantages. First it uses orders of magnitude smaller scale lengths than the maximal distance up to which the coding works properly. The largest grid scales measured experimentally are ∼3 m [23, 24] and extrapolations based on the dorso-ventral location of the recording electrodes within the entorhinal cortex extend to ∼10 m [13], a period still substantially smaller than the typical distances travelled daily by rodents (several hundreds of meters [25]) or bats (several kilometres, [26]; see also [27]).

Second, while the consequence of a module failure simple decreases the capacity of the system in the case of MA coding, it can have more dramatic effect in nested codes: Although malfunction of the largest or smallest module reduces either the capacity or the resolution of nested codes, respectively, the lack of intermediate modules functionally breaks the interaction between the remaining modules decreasing both the resolution and the capacity of the system in a disproportionate manner.

Third, once the scales are optimised for a given noise level, the coding range of nested grid codes does not depend on *δ*. Therefore, contrary to MA codes, it is not possible to increase the capacity by inserting more neurons into the same modules or by observing more grid cells from the same set of modules. Conversely, the functioning of the nested codes critically depends on accurate decoding of each module: If the readout neuron does not have access to enough presynaptic neurons from a given module, then the corresponding posterior becomes too wide leading to interference between the modules. This has similar consequences as the absence of the given module in nested codes. In contrast, in MA codes the coding properties remain similar for postsynaptic neurons receiving different number of synapses from different modules, although the coding range is the function of the precision available for the observer (Eq 9).

When encoding dynamic trajectories instead of static locations, the number of neurons required to participate in a given module decreases quadratically with the scale of the module, i.e., $n_{i}∼1/\alpha_{i}^{2}$ [8]. For example, if representing the position in the 2D space with some fixed accuracy with *α*_*i*_ = 0.2 m requires ∼ 4000 neurons then *α*_*j*_ = 2 m needs only ∼ 40 neurons. This scaling implies that the coding range of the nested grid system can be easily and parsimoniously extended by adding a new module with larger scale but containing only relatively few neurons. Although the relationship between the number of neurons in a module and its scale holds also for MA codes, the total number of neurons required to achieve similar coding range can be substantially smaller in nested codes.

Another consequence of dynamical coding is that the time constant of the readout has to be matched to the scale of the grid modules [8]. As the grid scale varies over a large range in the case of nested codes, the postsynaptic neuron has to integrate inputs from different grid cells with time constants ranging from 1 ms to 1 second [8]. In MA codes, the modules have similar scales and their outputs can be integrated with similar time constants.

Finally we note that nested coding and MA coding are not mutually exclusive: although they imply fundamentally different way of decoding the same positional information [7, 14], but both can be present in the same system. The MA code has a larger coding range if $c_{A}>\alpha \delta$ so it is favoured by small *α* (small differences between scales) and small *δ* (high accuracy). Even in this case locations within the largest grid scale can be decoded as in nested coding, while MA decoder is required beyond this distance.

### Planar grid cells

In the Methods we show that the coding capacity of two or higher dimensional grid cells depend on the same number theoretic properties, and therefore the results obtained in dimension one extend to planar or cubic grid cells as well [28], provided that the main axes of the different modules remain aligned with each other.

If the two dimensional grid modules are rotated compared to each other, then the scale choices which perform well will be different from the scales that are optimal for axis aligned modules. Consider for example that $\alpha =\sqrt{3}$, which is a relatively good choice for *M* = 2 (rightmost red circle in Fig 5a–5c), leads to cathastrophic interference at *ℓ* = *α* when the grids are rotated by 30°. Consequently, the incoherent reorientation of the grid modules during global remapping [13] renders the optimisation of the grid scales unfeasible. However, the main point of this paper is that we have shown analytically that almost all scale choices perform near optimally if the number of modules is high enough, which also applies for grid cells rotated uniformly at random relative to each other (Methods).

Moreover, the 2D grids does not need to show perfect triangular symmetry to achieve high capacity: environmental boundaries [29, 24] or non-euclidean geometry [30, 31] can distort the grid pattern, but as long as the distortion is coherent among modules, our theory applies unchanged. If the scales slightly vary on the long range, then our derivation based on the Diophantine approximations does not apply. However, our derivation stating exponential capacity for grid systems with many random scales (Methods) remains still valid.

### Optimization and robustness

The highly organised, regular patterns formed by the firing fields of grid cells suggest that the characteristics of the grids must be closely related to the computational function of these neurons: optimally representing and processing information about the spatial location of the animal [32, 33, 14, 4, 11]. Besides the general optimality of triangular grid-like firing fields for representing unbounded 2D space [28], recent theoretical work derived optimal scale ratio of successive grid modules in the case of nested coding [6, 7, 8].

These studies, using different assumptions, arrived at slightly different conclusions regarding the optimal value of *α*. Stemmler et al., [7] fixed both the coding range to *L*_max_ = 3 for a pair of grid modules with scales {1, *α*} and found that *α* = 3/2 minimises the ambiguity errors within that range. Mathis et al., [4] and Wei et al., [6] also fixed the coding range and minimised the number of neurons required to achieve a given resolution and provided both estimates for the maximal capacity of the grid cell coding system and a specific architecture (i.e., optimised nested codes) that achieves maximal efficiency. The optimal scale ratio for nested codes was found to depend both on the magnitude of the noise in the system and on the type of decoder [4, 6]. Rather than fixing the coding range, we were interested in grid codes that work for potentially unbounded environments and found a similar asymptotic capacity for MA codes using random grid scales. Although predictions derived from nested coding roughly agree with the average scale ratio observed in the entorhinal cortex [12, 13, 29], they do not explain the substantial amount of variability which characterises the data.

In our derivations we assumed that the decoding error of a given grid module is larger than *δ* with some small probability. Inaccurate decoding of a single grid module can lead to disproportionally high error in the position representation if the subsequent time frames are decoded independently [14, 9]. However, the chance of catastrophic ambiguity errors can be substantially reduced if a dynamical decoder combines prior information representing the predicted spatial position with the location encoded by the incoming grid cell spikes [34, 14, 8].

Our results based on the Diophantine approximations requires that the scale of the modules are set precisely, so that the phase of the different modules does not drift relative to each other (i.e., $\alpha_{i}\frac{\text{d}\phi_{i}}{\text{d}x}=\alpha_{j}\frac{\text{d}\phi_{j}}{\text{d}x}$). Although theoretical considerations suggest that drift can not be completely suppressed in a noisy neuronal system [35, 36], whether different grid modules respond coherently to distortions caused by environmental manipulations is not known [15, 29, 24]. The remarkable robustness of the grid system’s efficiency against the choice of the scale ratio suggests that grids with loosely set scale parameters could also obtain a similar performance. Indeed, our derivation using randomly selected grid scales does not require precisely set scale parameters yet it provides the asymptotically exponential capacity for the grid system.

The optimization principle assumes that substantial improvement in the performance of the system can be achieved with precise tuning of its parameters. In the present study we demonstrated that this is indeed the case in the absence of noise. However, even in this case, optimization would be almost unfeasible for three reasons. First, the coding range is an extremely irregular, discontinuous function of the scale parameter, making optimisation essentially a trial and error game. Second, a scale parameter that is optimal for a given number of modules is guaranteed to be inefficient when the number of modules is increased precluding the possibility of pairwise or modular optimization. Finally, the optimal grid scales depend on the rotation of the modules relative to each other, which can change independently during changes in the environment [13].

However, taking the variability of neuronal firing into account changes the picture dramatically. We demonstrated that when the coding accuracy of grid modules is limited by neuronal noise, the capacity of the system becomes surprisingly robust to the choice of the scale parameters making its optimization unnecessary. Note, that even if the grid periods are not optimized across modules, generating the regular, periodic firing fields of grid cells demands accurate integration of velocity inputs [37, 36] and repeated error correction [38, 35], both requiring the precise tuning of single neuron and network parameters within a given module. In conclusion, our study demonstrates that the capacity of the grid cell system is nearly optimal with randomly chosen grid scales, and, instead of accurate parameter tuning, the experimentally observed scales could reflect the combined effect of random fluctuations and a gradient in the cellular properties along the dorso-ventral axis of the entorhinal cortex [39, 40].

### Predictions

Our finding, that grid cells have an exponentially large coding range even with randomly chosen grid scales of similar magnitudes makes several important predictions. First, MA coding predicts that the coding range is substantially larger than the largest grid period. Since grid cells are likely to be involved in path integration [32, 41] this prediction could be tested by probing path integration abilities of rodents beyond distances of the largest grid period [42].

Second, in the case of MA coding, different modules have similar contributions to the coding range of the system. Therefore, the effect of targeted dMEC lesion (inactivating a single module, as in [43]) on the rat’s navigation behaviour would be largely independent of the actual location of the lesion (i.e., which module is inactivated).

Third, since the performance of the system is independent of the precise choice of the grid scales, we expect a large variability in the scale ratio of successive grid modules both within and across animals. This prediction is consistent with the experimental data available [12, 13, 29], although further statistical analysis would be required to specifically determine the distribution of scale ratios.

Finally, we predict that the performance of the system is not particularly sensitive to incoherent changes in the scale parameter of a subset of modules during e.g., global remapping induced by environmental changes [16]. It has been shown that under certain conditions simultaneously recorded grid cells respond coherently within a module and independently across modules to environmental distortions [13]. To test the prediction of our theory, the behavioural consequences of incoherent realignment across modules should be assessed and compared with the effects of environmental manipulations inducing coherent realignment [29] or coherent distortion in the shape of the grid pattern [24, 29, 30, 31].

## Methods

### Grid cells in the 2D plane

Consider a system *G*^2^ of planar grid cells with a set of scales A. Suppose that the axis of all modules are aligned and use the coordinate system

$$\begin{array}{c} B=\{\left[1,\;0\right],\;\left[\cos\left(60^{{}^{\circ}}\right),\;\sin\left(60^{{}^{\circ}}\right)\right]\}, \end{array}$$ (11)

which is naturally generated by the triangular lattice. To compare with consider the one dimensional grid cell system *G*^1^ which has the same number of modules with the same set of scales, and for which each module represents the position of the animal with the same relative precision. To achieve this, the two dimensional modules need squared as many cells, nevertheless they also able to distinguish between squared as many spatial positions within one period of the scale.

If *G*^2^ represents a planar position ${\left(x,y\right)}_{B}$ ambiguously, i.e., $\psi {\left(x,y\right)}_{B}\approx \psi {\left(0,0\right)}_{B}=0$, then clearly planar positions ${\left(x,0\right)}_{B}$ and ${\left(0,y\right)}_{B}$ are also represented ambiguously. Therefore, the corresponding one dimensional positions *x* and *y* are represented ambiguously by *G*^1^ as well. Conversely, if *z* is represented ambiguously by *G*^1^, then ${\left(z,0\right)}_{B}$, ${\left(0,z\right)}_{B}$ will be ambiguous in *G*^2^. Therefore, an ambiguity of position at a given distance from the origin in case of planar cells can be matched to an ambiguity at the same order of magnitude of distance in the one dimensional grid system, and vica versa. The above argument also shows that the same scale choices perform best for both one dimensional grid cells and two or higher dimensional ones when the axes are aligned with each other.

### Estimating the precision of a single module

We chose *α*_0_ = 1 and fix the resolution of the system to *δ* < *α*_0_ (defined below) and investigate its coding range. A formally identical system with a fixed coding range and optimised resolution can be achieved by appropriately rescaling the grid scales.

We numerically estimated the precision of position coding by a single module by first simulating the motion of the animal as a one dimensional Gaussian random walk:

$$\begin{array}{c} P\left(x_{t+1}|x_{t}\right)=N\left(x_{t},\Delta t\;D\right) \end{array}$$ (12)

with Δ*t* = 1 ms temporal resolution and *D* = 0.005 m^2^/s, which gives ≈ 5 cm displacement in 0.5 s [8]. We simulated the activity of *N* = [10, 300] grid cells from a single module. Grid cells had a circular tuning curve:

$$\begin{array}{c} r^{k}\left(x\right)=r_{\text{max}}{\left(\text{sin}\left(\frac{2\pi x}{2\lambda }-\phi^{k}\right)\right)}^{n}+r_{0} \end{array}$$ (13)

with the following parameters: *r*_max_ = 15 Hz, *r*_0_ = 0.1 Hz, λ = 0.25 m and *ϕ*^*k*^ chosen to uniformly cover the interval [0, 2*π*]. The power *n* = 22 was set to match the mean firing rate of the grid cells, 〈*r*(*x*)〉 = 2.5 Hz, to experimental data [16]. Larger (λ = 2.5 m) grid spacing was modelled by decreasing the speed of the animal by a factor of 10 (*D* = 0.00005 m^2^/s). The firing rate is shown in Fig 1b, right (olive).

Spike trains were generated as an inhomogeneous Poisson process with neurons conditionally independent given the simulated location:

$$P\left(s_{t}^{k}|x_{t}\right)=\text{Poisson}(\Delta t\;r^{k}\left(x_{t}\right))$$ (14)

Spikes of the neurons in module *i*, **s**_0:*t*,*i*_, represent the spatial location of the animal with error *δ α*_*i*_ (i.e., with the same *δ* phase error for all modules) which can be interpreted as the width of the (periodic) posterior probability distribution *P*(*x*|**s**_0:*t*,*i*_). For an ideal observer this posterior distribution quantifies how much a given spatial location is consistent with the observed spike pattern. The posterior distribution of the position was numerically calculated by recursive Bayesian filtering:

$$\begin{array}{c} P\left(x_{t}|s_{0:t}\right)\;\propto \;\prod_{k}P\left(s_{t}^{k}|x_{t}\right)\;\int P\left(x_{t-1}|s_{0:t-1}\right)P\left(x_{t}|x_{t-1}\right)\;dx_{t-1} \end{array}$$ (15)

The colormap in Fig 1b shows this posterior distribution with *N* = 50 cells and λ = 0.25 m.

Naturally, the width of the posterior depends on several factors, most importantly on the number of neurons observed in a given module and on the scale of the modules relative to the typical speed of the animal [8]. At each timestep the posterior distribution was fitted with a von Mises distribution with a location *μ*_*t*_ and a concentration parameter *κ*_*t*_. The width of the posterior relative to the grid scale was estimated as:

$$\begin{array}{c} \delta_{t}=\frac{\lambda }{2\pi \sqrt{\kappa_{t}}} \end{array}$$ (16)

For analytic tractability, we use a bounded noise model in the derivations assuming that the location decoded from the spikes of a module is within *δ* *α*_*i*_ distance from the true location. To be conservative, we chose *δ* to be the 99% of the empirical CDF of *δ*_*t*_. The largest *δ* = 0.12 was found with λ = 0.25 m and *N* = 10 cells. The smallest *δ* = 0.01 corresponds to the parameters λ = 2.5 m and *N* = 300 cells.

We assume that the modules are conditionally independent given the location of the animal, and hence position decoding, or representation, can be implemented by an ideal observer independently reading out the spikes, **s**_*i*_, emitted by the different modules: *P*(*x*|**s**) = ∏_*i*_ *P*(*x*|**s**_*i*_). When loosely talking about interference between the grid modules at a spatial point we refer to the interference between these periodic posterior distributions *P*(*x*|**s**_*i*_), i.e., all module posteriors being larger than 0 at a location different from the origin (Fig 1c).

### Interference at integer distances

Since we measure the distance in units of the smallest grid scale (*α*_0_ = 1), avoiding interference at integer distances from the origin also guarantees the absence of interference elsewhere, i.e., all positions in the interval [0, *L*] will be distinguishable by the grid code. Hence we loosely call *ϵ*(*ℓ*) defined in Eq 1 the phase difference, but note that it is the phase difference at integer distance *ℓ*. Indeed, if the grid code was ambiguous confusing spatial locations *x*_1_ and *x*_2_, then it would also confuse the origin with |*x*_1_ − *x*_2_| as well, since the phase differences of each module are the same between 0 and |*x*_1_ − *x*_2_| and between *x*_1_ and *x*_2_ (Fig 2b and 2c, right). But |*x*_1_ − *x*_2_| can be confused with the origin only if |*x*_1_ − *x*_2_| is an integer, that is a multiple of the smallest scale, 1. Note that this argument is correct only if the phase representation ambiguity of each module is independent of the actual position, which holds if we suppose that firing fields of cells from the same module are spaced evenly, which we do assume.

Graphically, interference between locations occurs when two segments of the phase curve come close to each other. Since the segments of the phase curve are parallel (Fig 2), and we started the phase curve in the origin, interference first occurs in the origin. Avoiding interference at the origin as much as possible at arbitrary distances thus also guarantees that the segments of the phase curve are separated from each other as much as possible, leading to a uniform coverage of the phase space [14].

### Definition of algebraic numbers

We call a real number *α* algebraic of order *n* (positive integer), if *n* is the least integer such that *α* is the root of a polynomial of degree *n* with integer coefficients. Algebraic numbers of order one are exactly the rational numbers. Another example is the golden ratio, *σ*, which is irrational, and is the root of *x*^2^ − *x* − 1, a integer polinomial of degree two. Therefore, *σ* is an algebraic number of order two.

### Information rate

Since we fixed the resolution, the capacity of the code is proportional to the coding range. Moreover, as the coding precision of the modules was the same, we assume that the population size of each module is approximately *N* for grid scales chosen randomly from a bounded interval. The information rate of the grid system, defined as the ratio of the logarithm of the capacity and the total number of conveyed bits [14] is

$$\rho \propto \frac{1}{\bar{r}NM}\text{log}{\left(\frac{c_{\alpha }}{\delta }\right)}^{M}$$ (17)

$$\propto \frac{1}{\bar{r}N}\log\frac{c_{\alpha }}{\delta }$$ (18)

$$\propto \frac{1}{\bar{r}N}\log\frac{c_{\alpha }\;\log N}{k}$$ (19)

where $\bar{r}$ is the average firing rate of a grid cell and in the third line we used that *δ* = *k*/log(*N*) [14]. Thus, the information rate is independent of the number of modules and increases with log *c*_*α*_.

For a geometric code with scale ratio *α* the optimal population size for dynamical decoding and constant *δ* decreases as $n_{i}=n_{0}/\lambda_{i}^{2}=n_{0}/\alpha^{2i}$ where λ_*i*_ = *α*^*i*^ is the scale of module *i* and *n*_0_ is the number of neurons in the first module [8]. In this case the total number of neurons in the population is

$$N=\sum_{i=0}^{M}n_{0}/\alpha^{2i}=\frac{n_{0}\;\alpha^{2}}{\alpha^{2}-1}$$ (20)

Since the total number of neurons does not grow linearly with the number of modules, the information rate becomes proportional to *M*:

$$\rho \propto M\frac{\alpha^{2}-1}{n_{0}\;\alpha^{2}\;\bar{r}}\log\frac{c_{\alpha }}{\delta }$$ (21)

Although a constraint on the minimal number of cells per module will limit the finite information rate to remain finite, Eq 21 emphasises that adding further modules with larger periods increases the efficiency of the grid system if the number of cells per module is set optimally for dynamical decoding [8]. Although a geometric progression of scales is consistent with both nested and MA codes, the information rate is higher for optimal nested codes since they maximise *α*.

### Interference with *M* modules I: Golden ratio is suboptimal

In this section we demonstrate that a set of grid cells with scale ratio (*α*) optimally chosen between pairs of successive grid modules is close to being pessimal for efficient space representation for *M* > 2. Such pairwise optimisation leads to a set of scales showing geometric progression with the scale ratio being *α*, i.e., [1, *α*, *α*^2^, …], which is consistent with the experimental data [10, 12, 23, 13]. The representation of the position becomes ambiguous if all modules show interference at the same location, i.e., the phase of all modules are very close to 0 at distance *ℓ* from the origin.

Consider for example the golden ratio *α* = *σ*, which is a second order algebraic number, i.e., it is the root of the integer coefficient polynomial *x*^2^ − *x* − 1. Therefore, the phase *ψ*_2_(*x*) = (*x* mod *σ*^2^)/*σ*^2^ of any spatial point *x* according to the third module can be simply expressed with the phase of the first two modules as

$$\psi_{2}\left(x\right)=\left[\psi_{0}\left(x\right)-\psi_{1}\left(x\right)\right]\;\text{mod}\;1.$$ (22)

To see this, consider that by the definition of the phases *ψ*_*i*_(*x*) when the animal is at distance *x* from the origin there are some integers *ℓ*, *k*_1_, *k*_2_ so that

$$\begin{array}{c} x=\ell +\psi_{0}\left(x\right)=\sigma \left(k_{1}+\psi_{1}\left(x\right)\right)=\sigma^{2}\left(k_{2}+\psi_{2}\left(x\right)\right). \end{array}$$

Using that *σ*^2^ − *σ* − 1 = 0 we get that

$$\begin{array}{c} \sigma^{2}\left(\ell +\psi_{0}\left(x\right)\right)-\sigma^{2}\left(k_{1}+\psi_{1}\left(x\right)\right)-\sigma^{2}\left(k_{2}+\psi_{2}\left(x\right)\right)=0. \end{array}$$

Rearranging terms yields

$$\begin{array}{cc} \psi_{2}\left(x\right) & =\ell -k_{1}-k_{2}+\psi_{0}\left(x\right)-\psi_{1}\left(x\right)\\ & =[\psi_{0}\left(x\right)-\psi_{1}\left(x\right)]\;\text{mod}\;1. \end{array}$$

In other words, the phase of the third module provides no additional information given the phase of the other two modules. In particular, if both *ψ*_0_(*x*) and *ψ*_1_(*x*) are close to 0 (Fig 6b), then so is *ψ*_2_(*x*) and hence the third module fails to resolve the ambiguity when the two first modules interfere. Similarly, if we have *n* grid cell modules with scales 1, *α*, …, *α*^*n*−1^ with *α* being an algebraic number of order *k* < *n*, then all of the *n* phases can be expressed by any *k* of them, leading to redundant and inefficient representation.

Clearly the same argument works not only for the powers of the golden ratio, but for powers of any algebraic number of order lower than the number of modules.

### Interference with *M* modules II

To derive the general solution for *M* grid modules, we consider a set of 1-dimensional grids with scales *α*_0_ = 1 < *α*_1_ < ⋯ < *α*_*M* −1_. Again, the interference between the modules can be expressed by the simultaneous Diophantine approximation of the vector $A=(\alpha_{1},\ldots ,\alpha_{M-1})$ using fractions of integers with the common numerator *ℓ*, i.e., *α*_*i*_ ≈ *ℓ*/*k*_*i*_. Importantly, a theorem by Dirichlet provides an upper bound on the efficiency of the approximation. Namely, for all (*M* − 1)-tuple of irrational numbers *α*_1_, …, *α*_*M* −1_ we have infinitely many collections of integers *k*_0_, *k*_1_, …, *k*_*M* −1_ (with *k*_0_ = *ℓ*), such that the approximation error defined as

$$\tilde{\epsilon }{}_{ij}\left(l\right)=\left|k_{i}\alpha_{i}-k_{j}\alpha_{j}\right|$$ (23)

is simultaneously smaller than the upper bound for all items in the tuple:

$$\begin{array}{c} {\tilde{\epsilon }}_{ij}\left(\ell \right)<\frac{\alpha_{i}+\alpha_{j}}{\ell^{1/(M-1)}}\;\left(\forall i,j=0,\ldots ,M-1,\;i\neq j\right). \end{array}$$ (24)

Note, that ${\tilde{\epsilon }}_{ij}$ differs from *ϵ* defined for two modules (Eq 2) as it is not normalised with *α*.

*Proof of* Eq 24. First we prove that any vector of irrationals can be approximated to the claimed order with rationals having the same denominator. Let $A=(\alpha_{1},\ldots ,\alpha_{n-1})$. To approximate A with rationals of denominator at most *Q* let us define the vectors $a_{j}=jA-\left\lfloor jA\right\rfloor$, *j* = 0, …, *Q*, where floor is understood coordinate-wise. Let us partition the unit cube [0, 1]^*n*−1^ into small cubes of side length *Q*^−1/(*n*−1)^, so that altogether we have *Q* of them. Since we have *Q* + 1 many **a**_*j*_-s each falling into [0, 1]^*n*−1^, hence there will be (at least) 2 of them falling into the same small cube, **a**_*k*_ and **a**_*l*_, say. Then

$$\left||k-l|A-|\left\lfloor kA\right\rfloor -\left\lfloor lA\right\rfloor ||\leq \right|a_{k}-a_{l}|\leq Q^{-1/(n-1)},$$

with the inequalities holding coordinate-wise. Therefore, because of |*k* − *l*| ≤ *Q*, A is approximable with denominator |*k* − *l*| and numerator (vector) |⌊kA⌋-⌊lA⌋| with error not exceeding |*k* − *l*|^−(1+1/(*n*−1))^. The desired statement follows then by simultaneously approximating the numbers 1/*α*_*i*_ with common denominator, which is also a simultaneous approximation of *α*_*i*_ with common numerator, which completes the proof.

For a set of grid scales *α*_*i*_ = *α*^*i*^ (*i* = 0, …, *M* − 1) where *α* is an algebraic number of degree *M*, there exists a maximal positive constant $c_{A}$, such that

$$\begin{array}{c} \tilde{\epsilon }\left(\ell \right)=\max_{i,j}\{\frac{1}{\alpha_{i}+\alpha_{j}}{\tilde{\epsilon }}_{ij}\left(\ell \right)\}>\frac{c_{A}}{\ell^{1/(M-1)}} \end{array}$$ (25)

holds, except for at most finitely many integers *ℓ*.

To see that Eq 25 holds, we start from the work of [44] (see also [45]) stating that powers of an algebraic number are badly simultaneously approximable with common denominator in the following sense. Let *β* be an algebraic number of order *M*. There exists *c*_*β*_ > 0 such that for all integer *ℓ*, *k*_*i*_ there is *i* ∈ {1, …, *M* − 1} for which

$$\begin{array}{c} |\beta^{i}\ell -k_{i}|>\frac{c_{\beta }}{\ell^{1/(M-1)}}. \end{array}$$

*Derivation of* Eq 25. Our goal is to give a lower bound on |*α*^*i*^ *k*_*i*_ − *α*^*j*^ *k*_*j*_|, where *α* is algebraic of order *M*, 0 ≤ *i*, *j* ≤ *M* − 1. Without loss of generality suppose that *i* < *j*.

$$\begin{array}{c} |\alpha^{i}k_{i}-\alpha^{j}k_{j}\left|=\right|\alpha^{i-j}k_{i}-k_{j}|\alpha^{j}>\alpha^{j}\frac{c_{A}}{k_{i}^{1/(M-1)}}. \end{array}$$

Now the fact that *k*_*i*_ ∼ *ℓ*/*α*^*i*^ implies Eq 25 if $c_{A}>0$ is chosen appropriately.

The position representation is unambiguous if there is at least one pair of modules for which the phase difference is larger than the threshold set by the noise, i.e., ${\tilde{\epsilon }}_{i,j}\left(\ell \right)>\delta \left(\alpha_{i}+\alpha_{j}\right)$ which holds if

$$\begin{array}{c} \delta <\frac{c_{A}}{\ell^{1/(M-1)}} \end{array}$$ (26)

From here, the critical distance *L*_max_ up to which coding is unambiguous can be expressed as (cf. Eq 9):

$$\begin{array}{c} L_{\text{max}}≔{\left(\frac{c_{A}}{\delta }\right)}^{M-1}, \end{array}$$ (27)

for all *δ* which is small enough.

To directly compare the capacity of the MA grid cell system derived in Eq 9 with previous estimates for nested coding [4, 6], we also calculate *N*_max_, the number of distinguishable spatial phases:

$$N_{\text{max}}≔\frac{L_{\text{max}}}{2\delta }=\frac{1}{2c_{A}}{\left(\frac{c_{A}}{\delta }\right)}^{M}$$ (28)

Efficient coding with nested modules requires that *α*_*i*_ = *r*^*i*^ with 0 ≤ *i* ≤ *M* − 1 and *r* being the scale ratio with fixed relative uncertainty of modules 2*δ* = 1/*r* [6]. The position of the animal can be determined at precision 1/*r* without ambiguity if the animal is restricted to move in an environment with the size identical to the scale of the largest module, *r*^*M*−1^. In this case the number of distinguishable spatial phases is $r^{M}={\left(\frac{1/2}{\delta }\right)}^{M}$, which is identical to the capacity we found for non-nested coding when $c_{A}=0.5$ (Eq 28).

### Coding is unambiguous up to exponential distance in the number of modules

To derive Eq 27 we first show that interference of the grid representation is equivalent to pairwise interference between all pairs of modules. To test unambiguity of coding note that the place at distance *x* from the origin is confusable with 0 if for all *i* = 0, …, *M* − 1 there exists an integer *k*_*i*_ such that

$$\begin{array}{c} |k_{i}\alpha_{i}\;-\;x|<\alpha_{i}\delta , \end{array}$$ (29)

where *δ* is the relative uncertainty of modules. It turns out that, as for *M* = 2, there is no need to consider all *x* ∈ [0, *L*_max_], it is enough to care with integers:

**Claim**. *There exists x* ∈ [0, *L*_max_] *for which* Eq 29 *holds for all i exactly when the following pairwise interference occurs between all modules*:

$$\begin{array}{c} |k_{i}\alpha_{i}-k_{j}\alpha_{j}|<\left(\alpha_{i}+\alpha_{j}\right)\delta \end{array}$$ (30)

*for all i, j with some integers k_i_ (i* = 0, …, *M* − 1*) such that* 0 < *k*_*i*_ *α*_*i*_ ≤ *L*_max_.

*Proof*. Let us fix *k*_*i*_, *i* = 0, …, *M* − 1. Pairwise interference means that there is a point *x*_*i*,*j*_ in the intersection of (*k*_*i*_ *α*_*i*_ − *α*_*i*_ *δ*, *k*_*i*_ *α*_*i*_ + *α*_*i*_ *δ*) = (*a*_*i*_, *b*_*i*_) and (*k*_*j*_ *α*_*j*_ − *α*_*j*_ *δ*, *k*_*j*_ *α*_*j*_ + *α*_*j*_ *δ*) = (*a*_*j*_, *b*_*j*_). Due to the topology of the line, it is easy to see by induction that the intersection of all such intervals is nonempty and hence one can chose *x*_*i*,*j*_ = *x*. The statement is obvious for *M* = 2. Now suppose that the intersection $\cap_{i=0}^{n}\left(a_{i},b_{i}\right)\neq \emptyset$. Then it is the interval (*a*, *b*) with

$$\begin{array}{c} a=\max_{i=0,\ldots ,n}a_{i}\;\text{and}\;b=\min_{i=0,\ldots ,n}b_{i}. \end{array}$$

If (*a*_*n*+1_, *b*_*n*+1_) intersects (*a*_*i*_, *b*_*i*_), then both *a*_*n*+1_ < *b*_*i*_ and *b*_*n*+1_ > *a*_*i*_, and therefore *a*_*n*+1_ < *b* and *b*_*n*+1_ > *a*, which completes the induction. Therefore Eq 29 implies Eq 30. The other direction is immediate.

Now using the above Claim Equation Eq 27 easily follows by rearranging Eq 25.

### Asymptotic capacity of the random grid cell system

Let us fix the relative uncertainty of modules *δ* < 1/2 and a number *α*_max_ > (1 + *δ*)/(1 − *δ*). We show that if scales *α*_1_, *α*_2_, … are drawn uniformly at random from [1, *α*_max_], independently of each other, then for any *δ* < *ζ* < 1/2 the representation with *M* modules having scales *α*_1_, *α*_2_, …, *α*_*M*_ is unambiguous in every spatial position *x* > 0 up to

$$\begin{array}{c} X_{\text{max}}≔{\left(\frac{\zeta }{\delta }\right)}^{M-1} \end{array}$$ (31)

with probability of order 1 − (2*ζ*)^*M*^ as *M* → ∞.

Here *ζ* is the analog of $c_{A}$ which characterises the capacity of a particular grid cell system. As we will see, the convergence holds for any *ζ* < 1/2, but the speed of the convergence depends on *ζ*: higher efficiency is guaranteed to be achieved only for larger number of modules.

*Proof:* Let *α*_1_, *α*_2_, … be independent random variables distributed uniformly on [1, *α*_max_]. Let *x* be a spatial point and let ${\tilde{\psi }}_{i}={\tilde{\psi }}_{i}\left(x,\alpha_{i}\right)$ denote the phase of module *i* (with scale *α*_*i*_) at *x*, that is

$$\begin{array}{c} {\tilde{\psi }}_{i}≔\left(x\;\text{mod}\;\alpha_{i}\right)/\alpha_{i}=x/\alpha_{i}\;\text{mod}\;1=x/\alpha_{i}-\lfloor \frac{x}{\alpha_{i}}\rfloor \in \left[0,1\right]. \end{array}$$

Note that for fixed *x* the distribution of phases ${\tilde{\psi }}_{i}$ are independent of each other since the *α*-s are independent. We also use the notation *p*_1_(*x*) for the probability that the phase ${\tilde{\psi }}_{i}$ is (almost) indistinguishable from 0, defined in the following way:

$$\begin{array}{c} p_{1}\left(x\right)=P_{\alpha_{i}}({\tilde{\psi }}_{i}\in [0,(1+\varepsilon )\delta ]\cup [(1-(1+\varepsilon )\delta ),1]\;|\;x]. \end{array}$$

where *ε* > 0 is determined later. It is easy to see that *p*_1_(*x*) does not depend on *i*, i.e., it is the same for all modules. Moreover, the distribution of ${\tilde{\psi }}_{i}$ converges to uniform as the distance increases, in particular lim_*x* → ∞_ *p*_1_(*x*) = 2(1 + *ε*)*δ*. The convergence of this distribution to the uniform is a key observation that remains true even in higher dimensions with uniform random rotations or in case of slight variation of the grid scales on the long range. Hence there exists a critical distance, *x*_0_ = *x*_0_(*δ*, *ε*) for which all *x* > *x*_0_ we have |*p*_1_(*x*) − 2(1 + *ε*)*δ*| ≤ *δε*. Therefore, for *x* > *x*_0_ we have

$$\begin{array}{c} p_{1}\left(x\right)\leq \left(2+3\varepsilon \right)\delta . \end{array}$$ (32)

It also implies a bound on the probability of interference of many modules at a given point *x*. If we consider *M* modules with scales drawn uniformly at random from [1, *α*_max_] and independently of each other, then by Eq 32 for *x* > *x*_0_ the probability of all phases being close to 0 is

$$\begin{array}{cc} p_{M}\left(x\right) & =\mathbb{P}(\left(\forall i\leq M\right)\;{\tilde{\psi }}_{i}\in \left(0,\left(1+\varepsilon \right)\delta \right)\cup \left(\left(1-\left(1+\varepsilon \right)\delta \right),1\right)\;|\;x)\\ & =p_{1}{\left(x\right)}^{M}\leq {\left(\left(2+3\varepsilon \right)\delta \right)}^{M}, \end{array}$$ (33)

that is, *p*_*M*_(*x*) is exponentially small in *M*.

There remains to estimate the probability of interference of many modules anywhere up to a maximally allowed spatial distance. Our goal is to show that

$$\begin{array}{c} P(\left(\exists x<X_{\text{max}}\right)\left(\forall i\leq M\right)\;{\tilde{\psi }}_{i}\left(x\right)\in (0,\delta )\cup ((1-\delta ),1))\to 0 \end{array}$$ (34)

as *M* → ∞, where $X_{\text{max}}={\left(\frac{\zeta }{\delta }\right)}^{M-1}$, as in Eq 31. Note, that satisfying Eq 34 is not trivial, since *X*_max_ increases exponentially with *M*.

There is no need to investigate all *x* < *X*_max_, it is enough to show, that there is no interference on a set which is dense enough in [0, *X*_max_] in the stronger sense of Eq 33. Indeed, let *Y* be an *ε* dense set in [0, *X*_max_] with at most 2*X*_max_/*ε* elements. Then

$$\begin{array}{l} \left\{\left(\exists x<X_{\text{max}})(\forall i\leq M){\tilde{\psi }}_{i}(x)\in (0,\delta )\cup ((1-\delta ),1\right)\right\}\\ \Rightarrow \left\{\left(\exists x\in Y)(\forall i\leq M){\tilde{\psi }}_{i}(x)\in (0,(1+\varepsilon )\delta )\cup ((1-(1+\varepsilon )\delta ),1\right)\right\} \end{array}$$

where we used the fact that the $\frac{d}{dx}{\tilde{\psi }}_{i}=\frac{1}{\alpha_{i}}<1$ since *α*_*i*_ was chosen from the interval [1, *α*_max_]. The corresponding inequality for the probabilities of these events is

$$\begin{array}{c} \begin{array}{c} \mathbb{P}\left((\exists x<X_{\text{max}})\left(\forall i\leq M\right)\;{\tilde{\psi }}_{i}\left(x\right)\in \left(0,\delta \right)\cup \left(\left(1-\delta \right),1\right)\right) \end{array}\\ \leq \mathbb{P}\left(\left(\exists x\in Y\right)\left(\forall i\leq M\right)\;{\tilde{\psi }}_{i}\left(x\right)\in \left(0,\left(1+\varepsilon \right)\delta \right)\cup \left(\left(1-\left(1+\varepsilon \right)\delta \right),1\right)\right). \end{array}$$

Now for these finitely many points *x* ∈ *Y* we can use Eq 33 one by one, if *x* > *x*_0_:

$$\begin{array}{cc} \mathbb{P}((\exists x_{0}<x\in Y)\left(\forall i\leq M\right)\;{\tilde{\psi }}_{i}\left(x\right) & \in \left(0,\left(1+\varepsilon \right)\delta \right)\cup \left(\left(1-\left(1+\varepsilon \right)\delta \right),1\right))\\ & \leq {\left(\left(2+3\varepsilon \right)\delta \right)}^{M}2X_{\text{max}}/\varepsilon \\ & =\frac{2}{\varepsilon }{\left(\left(2+3\varepsilon \right)\delta \right)}^{M}{\left(\frac{\zeta }{\delta }\right)}^{M-1}\\ & <\frac{1}{\varepsilon \zeta }{\left(\zeta \left(2+3\varepsilon \right)\right)}^{M}\to 0 \end{array}$$

if $\varepsilon <\frac{1-2\zeta }{3\zeta }$, which we assume, where in the first inequality we used Eq 33 and union bound, and then in the second one that $X_{\text{max}}={\left(\frac{\zeta }{\delta }\right)}^{M-1}$. We have to remark that interference in different spatial points is not independent of each other, but union bound works even in that case.

There remains to show that the grid cell representation works up to *x*_0_. Clearly there is no ambiguity up to *x* = 1 + *δ*. To estimate the probability

$$\begin{array}{c} P(\left(\exists x_{0}\geq x\in Y\right)\left(\forall i\leq M\right)\;{\tilde{\psi }}_{i}\left(x\right)\in (0,(1+\varepsilon )\delta )\cup ((1-(1+\varepsilon )\delta ),1)) \end{array}$$ (35)

we first have to observe that the cardinality of *Y* ∩ [1 + *δ*, *x*_0_] is independent of *M*. Therefore to guarantee that the probability in Eq 35 goes to 0 we need to show that for all 1 + *δ* ≤ *x* ≤ *x*_0_ there is a scale *α* ∈ [1, *A*] which is able to distinguish *x* from the origin, that is *α* such that

$$\begin{array}{c} \tilde{\psi }\left(\alpha \right)=\left(x/\alpha \;\mathrm{mod}\;1\right)\in \left[\delta ,1-\delta \right]. \end{array}$$

This is so because *x*/*α* is monotonically decreasing in *α* and because

$$\begin{array}{c} x/1-x/A\geq x(2\delta /(1+\delta ))\geq 2\delta , \end{array}$$

where we used that *α*_max_ > (1 + *δ*)/(1 − *δ*) and *x* > 1 + *δ*. Therefore $\tilde{\psi }\left(\alpha \right)$ can not lay in [0, *δ*] ∪ [1 − *δ*, 1] for all *α* ∈ [1, *α*_max_].

### Numerical estimation of the $c_{A}$ with *M* modules

The constant $c_{A}$ (and *c*_*α*_) is well defined only for algebraic numbers, but can also be estimated for real numbers from the scaling of the phase difference with distance using numerical simulations. As $c_{A}$ is defined asymptotically (Eq 25), in order to estimate it numerically we need an approximation of it for finite distances. An alternative definition of $c_{A}$ (equivalent with Eq 25) is

$$\begin{array}{c} c_{A}=\underset{\ell \to \infty }{lim inf}{\hat{\epsilon }}_{A}\left(\ell \right), \end{array}$$ (36)

where ${\hat{\epsilon }}_{A}\left(\ell \right)$ is defined by

$$\begin{array}{c} {\hat{\epsilon }}_{A}\left(\ell \right)=\min_{K,k_{0}=\ell }\max_{i,j}\{|\alpha_{i}k_{i}-\alpha_{j}k_{j}|/\left(\alpha_{i}+\alpha_{j}\right)\}\ell^{1/(M-1)}, \end{array}$$ (37)

where $K=(k_{1},\ldots ,k_{M-1})$. Intuitively, to find the magnitude of interference at location *ℓ*, for all possible values of K we first select the maximum phase difference in the set and then choose the set with the smallest maximum. From the plots Figs 4 and 7 it is clear that the naive way of approximating $c_{A}$ with $c_{A}\left(\ell \right)$ for some large *ℓ* is not a good idea, as $c_{A}\left(\ell \right)$ may vary heavily with *ℓ*, especially for non-algebraic scale ratios. Note, that the calculation of ${\hat{c}}_{\alpha }$ is a special case of ${\hat{c}}_{A}$ with *M* = 2.

To estimate coding efficiency in the presence of noise we are mostly interested in the above infemum when *ℓ* is such that the phase difference ${\hat{\epsilon }}_{A}\left(\ell \right)/\ell^{1/(M-1)}$ is close to the precision *δ* of the modules. It motivates to investigate the (numerically computable) minimum

$$\begin{array}{c} {\hat{c}}_{A}\left(\delta_{1},\delta_{2}\right)=\min\{{\hat{\epsilon }}_{A}\left(\ell \right)\;|\;\ell_{2}\left(\delta_{2}\right)\leq \ell \leq \ell_{1}\left(\delta_{1}\right)\} \end{array}$$

for some pair *δ*_1_ < *δ*_2_, where *ℓ*_2_ is so that for all *ℓ* ≥ *ℓ*_2_ we have ${\hat{\epsilon }}_{A}\left(\ell \right)/\ell^{1/(M-1)}<\delta_{2}$ and *ℓ*_1_ is the smallest *ℓ* so that ${\hat{\epsilon }}_{A}\left(\ell \right)/\ell^{1/(M-1)}<\delta_{1}$.

### Tools for the numerical investigation of Diophantine approximation

A common and natural way to numerically investigate Diophantine approximation is using lattice reduction [46]. By lattice we mean a subset L of $R^{d}$ defined by some vectors $v_{1},\ldots ,v_{m}\in R^{d}$, *m* ≤ *d* so that

$$\begin{array}{c} L=\{w=\sum_{i=1}^{m}b_{i}v_{i}|b_{i}\in Z\}. \end{array}$$

**Fig 8:**
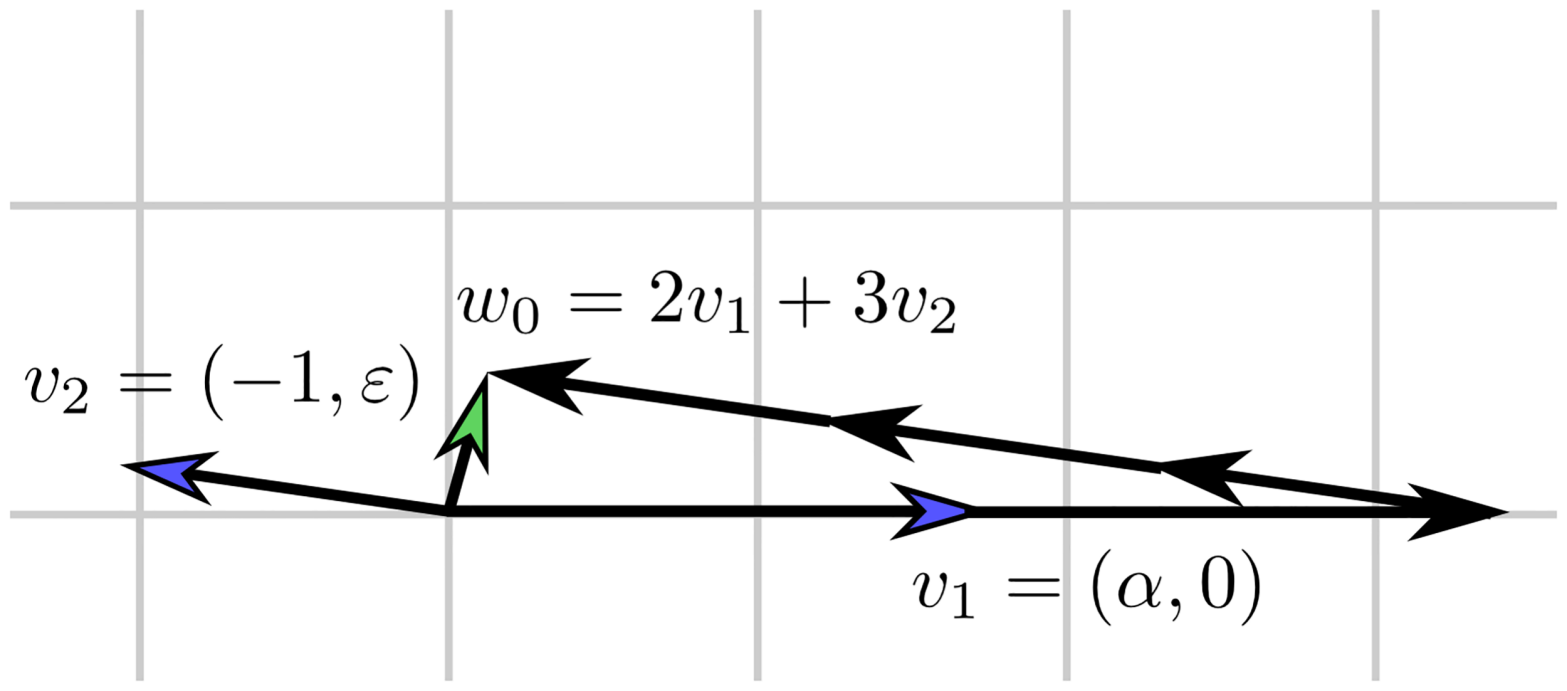
Which element of the lattice generated by the above two blue headed vectors is closest to the origin? Or in other words, what is the shortest nonzero vector which can be obtained as an integer coefficient linear combination of the above vectors?

Given a lattice L, a classical computational problem is to find the shortest non-zero vector of it (Fig 8). In the followings we show how Diophantine approximation of a vector (*α*_1_, …, *α*_*n*_) can be investigated with the help of finding shortest vectors of appropriately chosen lattices.

Let us first consider a simple example. Let the lattice L be defined by the rows of the matrix

$$\begin{array}{c} V=[\begin{array}{c} v_{1}\\ \vdots \\ v_{n+1} \end{array}]≔[\begin{array}{ccccc} \alpha_{1} & 0 & \ldots & 0 & 0\\ 0 & \alpha_{2} & \ldots & 0 & 0\\ \vdots & \vdots & \ddots & \vdots & \vdots \\ 0 & 0 & \ldots & \alpha_{n} & 0\\ -1 & -1 & \ldots & -1 & \varepsilon \end{array}], \end{array}$$

where *ε* > 0. For all *ε* which is small enough the shortest vector $w_{0}=\sum_{i=1}^{n+1}b_{i}v_{i}$ of L corresponds to a simultaneous Diophantine approximation of (*α*_1_, …, *α*_*n*_) with the common numerator *b*_*n*+1_ and denominators *b*_*i*_, *i* = 1, …, *n*. The parameter *ε* can be considered as a penalty term: the smaller this term the bigger the numerator can be.

When speaking about shortest vectors we need to specify the norm with respect to which vectors are compared. Here we are looking for the largest phase difference between the modules so we use supremum norm (Eq 25). The shortest vector in supremum norm of the lattice defined by *V* is an approximation so that

$$\begin{array}{c} \max\{b_{n+1}\varepsilon ,\;\max_{i}\{|b_{n+1}-b_{i}\alpha_{i}|\} \end{array}$$

is as small as possible. By this we can compute what is the maximal phase difference between the module with scale 1 and all other modules up to distance *b*_*n*+1_.

Remember that according to Eq 25 we are searching for an approximation minimizing

$$\begin{array}{c} \max\{b_{n+1}\varepsilon ,\;\max_{i,j}\{|\alpha_{i}k_{i}-\alpha_{j}k_{j}|/\left(\alpha_{i}+\alpha_{j}\right)\}\ell^{1/(M-1)}\}. \end{array}$$ (38)

Similarly to the previous example, it can be done simply by dividing columns *i*, *i* = 1, …, *n* of *V* by (1 + *α*_*i*_), and by adding some more columns of similar form which refer to interference between modules *i* and *j*. For example, for *n* = 3 the shortest (in sup norm) element of the lattice generated by the rows of the following matrix gives an approximation minimizing Eq 38:

$$[\begin{array}{ccccccc} \frac{\alpha_{1}}{1+\alpha_{1}} & 0 & 0 & \frac{\alpha_{1}/\alpha_{3}}{\alpha_{1}+\alpha_{3}} & 0 & \frac{\alpha_{1}/\alpha_{2}}{\alpha_{1}+\alpha_{2}} & 0\\ 0 & \frac{\alpha_{2}}{1+\alpha_{2}} & 0 & 0 & \frac{\alpha_{2}/\alpha_{3}}{\alpha_{2}+\alpha_{3}} & \frac{-1}{\alpha_{1}+\alpha_{2}} & 0\\ 0 & 0 & \frac{\alpha_{3}}{1+\alpha_{3}} & \frac{-1}{\alpha_{1}+\alpha_{3}} & \frac{-1}{\alpha_{2}+\alpha_{3}} & 0 & 0\\ \frac{-1}{1+\alpha_{1}} & \frac{-1}{1+\alpha_{2}} & \frac{-1}{1+\alpha_{3}} & 0 & 0 & 0 & \varepsilon \end{array}].$$

In this way maximal interference in the grid cell system can be computed numerically as shortest vectors of some lattices in supremum norm. Finding this shortest vector is an integer linear programming (ILP) problem, which in general is an NP-hard computational problem, and can be solved by e.g. a branch and bound algorithm [47]. There are also efficient methods which find approximation solutions in polynomial time, such as the LLL algorithm due to Lenstra, Lenstra and Lovász [46].

The LLL algorithm finds not only a short vector of a lattice, but also another basis of it which consists of short and nearly orthogonal vectors in the *L*^2^ norm, a so called LLL reduced basis. The error made by the LLL algorithm is too high to precisely compute the constant terms in Eq 27, and therefore we could not rely only on this algorithm. Nevertheless, compared to the ILP solution, we could significantly speed up our computations by first applying the LLL algorithm to find an approximate solution (and a reduced lattice), and then an ILP solver on this LLL reduced basis, which could find nontrivial optimal solutions very efficiently if started from this input.
